# Impact of physicochemical parameters of *Aedes aegypti* breeding habitats on mosquito productivity and the size of emerged adult mosquitoes in Ouagadougou City, Burkina Faso

**DOI:** 10.1186/s13071-022-05558-3

**Published:** 2022-12-20

**Authors:** Wendegoudi Mathias Ouédraogo, Kobié Hyacinthe Toé, Aboubacar Sombié, Mafalda Viana, Clarisse Bougouma, Antoine Sanon, David Weetman, Philip J. McCall, Hirotaka Kanuka, Athanase Badolo

**Affiliations:** 1 Laboratoire d’Entomologie Fondamentale et Appliquée, Université Joseph Ki-Zerbo, Ouagadougou, Burkina Faso; 2 Programme National de Lutte Contre Les Maladies Tropicales Négligées, Ministère de la Santé, Ouagadougou, Burkina Faso; 3 Institut National de Santé Publique, Centre National de Recherche et de Formation sur le Paludisme, Ouagadougou, Burkina Faso; 4 School of Biodiversity, One Health and Veterinary Medicine, University of Glasgow, Glasgow, UK; 5 Department of Vector Biology, Liverpool School of Tropical Medicine, Liverpool, UK; 6 Department of Tropical Medicine, The Jikei University School of Medicine, Tokyo, Japan

**Keywords:** *Ae. aegypti*, Breeding sites, Larvae, Pupae, *Stegomyia* index, Body size, Dengue

## Abstract

**Background:**

Outbreaks of dengue fever caused by viruses transmitted by *Aedes aegypti* mosquitoes are repeated occurrences in West Africa. In recent years, Burkina Faso has experienced major dengue outbreaks, most notably in 2016 and 2017 when 80% of cases were recorded in Ouagadougou City (Central Health Region). In order to better understand the ecology of this vector and to provide information for use in developing control measures, a study on the characteristics of *Aedes* container breeding sites and the productivity of such sites, as measured by the abundance of immature stages and resultant adult body size, was undertaken in three health districts (Baskuy, Bogodogo and Nongremassom) of Ouagadougou.

**Methods:**

Adult mosquitoes were collected indoors and outdoors in 643 households during the rainy season from August to October 2018. The presence of water containers was systematically recorded and the containers examined for the presence or absence of larvae. Characteristics of the container breeding sites, including size of the container and temperature, pH and conductivity of the water contained within, were recorded as well as the volume of water. Traditional *Stegomyia* indices were calculated as quantitative indicators of the risk of dengue outbreaks; generalised mixed models were fitted to larval and pupal densities, and the contribution of each covariate to the model was evaluated by the *Z*-value and associated *P*-value.

**Results:**

A total of 1061 container breeding sites were inspected, of which 760 contained immature stages of *Ae. aegypti* (‘positive’ containers). The most frequent container breeding sites found in each health district were tyres and both medium (buckets/cans/pots) and large (bins/barrels/drums) containers; these containers were also the most productive larval habitats and the types that most frequently tested positive. Of the* Stegomyia* indices, the Breteau, House and Container indices exceeded WHO dengue risk thresholds. Generalised linear mixed models showed that larval and pupal abundances were associated with container type, physicochemical characteristics of the water and collection month, but there were significant differences among container types and among health districts. *Aedes aegypti* body size was positively associated with type and diameter of the container, as well as with electrical conductivity of the water, and negatively associated with pH and temperature of the water and with the level of exposure of the container to sunlight.

**Conclusion:**

This study provides data on putative determinants of the productivity of habitats regarding *Ae. aegypti* immature stages. These data are useful to better understand *Ae. aegypti* proliferation. The results suggest that identifying and targeting the most productive container breeding sites could contribute to dengue vector control strategies in Burkina Faso.

**Graphical Abstract:**

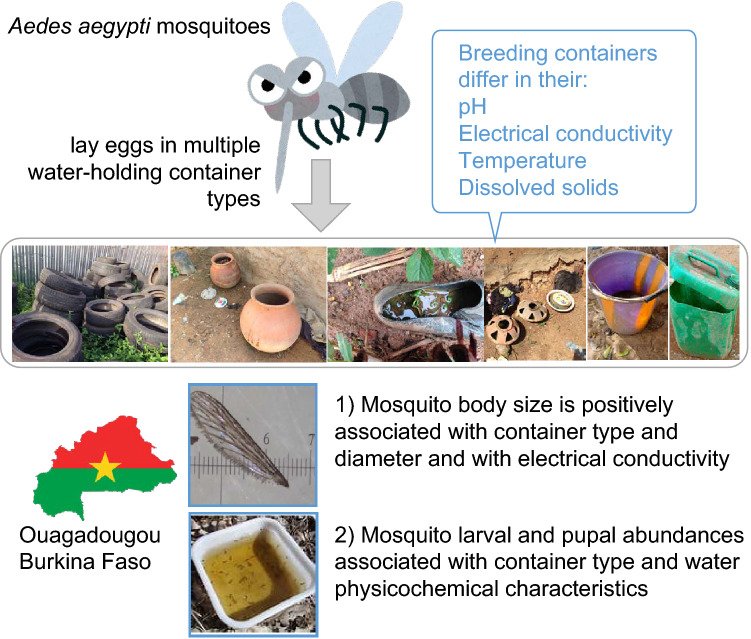

**Supplementary Information:**

The online version contains supplementary material available at 10.1186/s13071-022-05558-3.

## Background

*Aedes aegypti* is the most prolific vector involved in the transmission of important human arboviruses, including chikungunya virus (CHIKV), dengue virus (DENV), yellow fever virus (YFV) and Zika virus (ZIKV) [1, 2]. The worldwide emergence of arboviruses, such as DENV, represents a major public health concern on the African continent where 34 countries are considered to be endemic for dengue [3]. Successive dengue outbreaks have been recorded since 2013 in the city of Ouagadougou (Burkina Faso) [4–7], with the outbreak of 2016–2017 being the most important to date [8, 9].

*Aedes aegypti* is highly anthropophilic [10, 11], well adapted to human environments and breeds in diverse artificial and domestic containers [12, 13]. The typology and productivity of these containers can vary between countries but also between localities within the same country [13–16]. A recent study in Burkina Faso showed that tyres and small containers were the most abundant breeding containers in urban localities but that drums were the most abundant breeding containers in the peri-urban and rural localities due to the need to store water, a consequence of the absence of a piped water supply in these localities [17]. Productivity, as measured by the number of mosquito pupae, also varied across containers and localities. These results partly reflect wider findings across African countries, in which used tyres and discarded containers have been reported to be the most predominant and productive water-holding container breeding sites for mosquitoes [18–20].

In the absence of specific antiviral treatments and effective vaccines, entomological surveillance and vector control remain the most effective strategy for dengue control [21, 22]. Given the high insecticide resistance in adult *Ae. aegypti* mosquitoes in Burkina Faso [23, 24], larval source management may present a sustainable control strategy [25]. An understanding of how the type and characteristics of container breeding sites contribute to *Stegomyia* indices and affect mosquito life-history traits is essential to establish locale-specific evidence-based surveillance and effective vector control for prevention and outbreak management.

Adult life-history traits of holometabolous insects are shaped during larval development with carry-over effects on subsequent fitness of adults and pathogen-vector interactions [26]. The development of *Ae. aegypti* immature stages depends on their the water characteristics of the container, including nutrient content, water volume, pH, temperature, conductivity, dissolved oxygen, among others. These parameters in turn can have effects on adult life-history traits, including body size, longevity, vectorial capacity and ultimately disease epidemiology. The characteristics of the water, including the pH, salinity and total dissolved solids, have also been found to be positively correlated with immature densities [27, 28], while dissolved oxygen and water container type predict the presence—but not necessarily the density—of immatures.

Rearing environment has been shown to have effects on mosquito body size; for example, *Ae. aegypti* reared under optimum laboratory conditions have larger and less variable body size compared to their field-reared cohorts [29]. Food and temperature during mosquito larval development have been shown to have contrasting effects on adult body size, with body size decreasing with increasing temperature and decreasing food levels [30]. In the same study, larger adult males lived longer than smaller ones, but no size effect was observed for females [30], as also documented in another study for daily survival rate [31]. Larval environment may also differentially impact stages of DENV-2 infection (i.e. midgut, dissemination or saliva) via carry-over effects impacting adults in *Aedes albopictus* [32].

Blood-feeding may also be impacted by larval environment: duration of the gonotrophic cycle was reported to be shorter in larger indoor-adapted *Ae. aegypti* females than in smaller, outdoor-adapted females [33]. While the length of the gonotrophic cycle influences vectorial capacity, the overall impact of larval conditions on the vectorial capacity of adult mosquito populations is likely to be more complex [34, 35]. Nevertheless, investigating the effect of breeding site characteristics on larval abundance and adult body size can provide useful data for understanding the ecology of *Aedes* populations and targeting productive breeding sites.

In the study reported here, we investigated the impact of characteristics of breeding containers, including physicochemical parameters of the water contained in these containers, on the *Stegomyia* indices, larval abundance and adult body size in three health districts of Ouagadougou City, Burkina Faso.

## Methods

### Study area

**Fig. 1:**
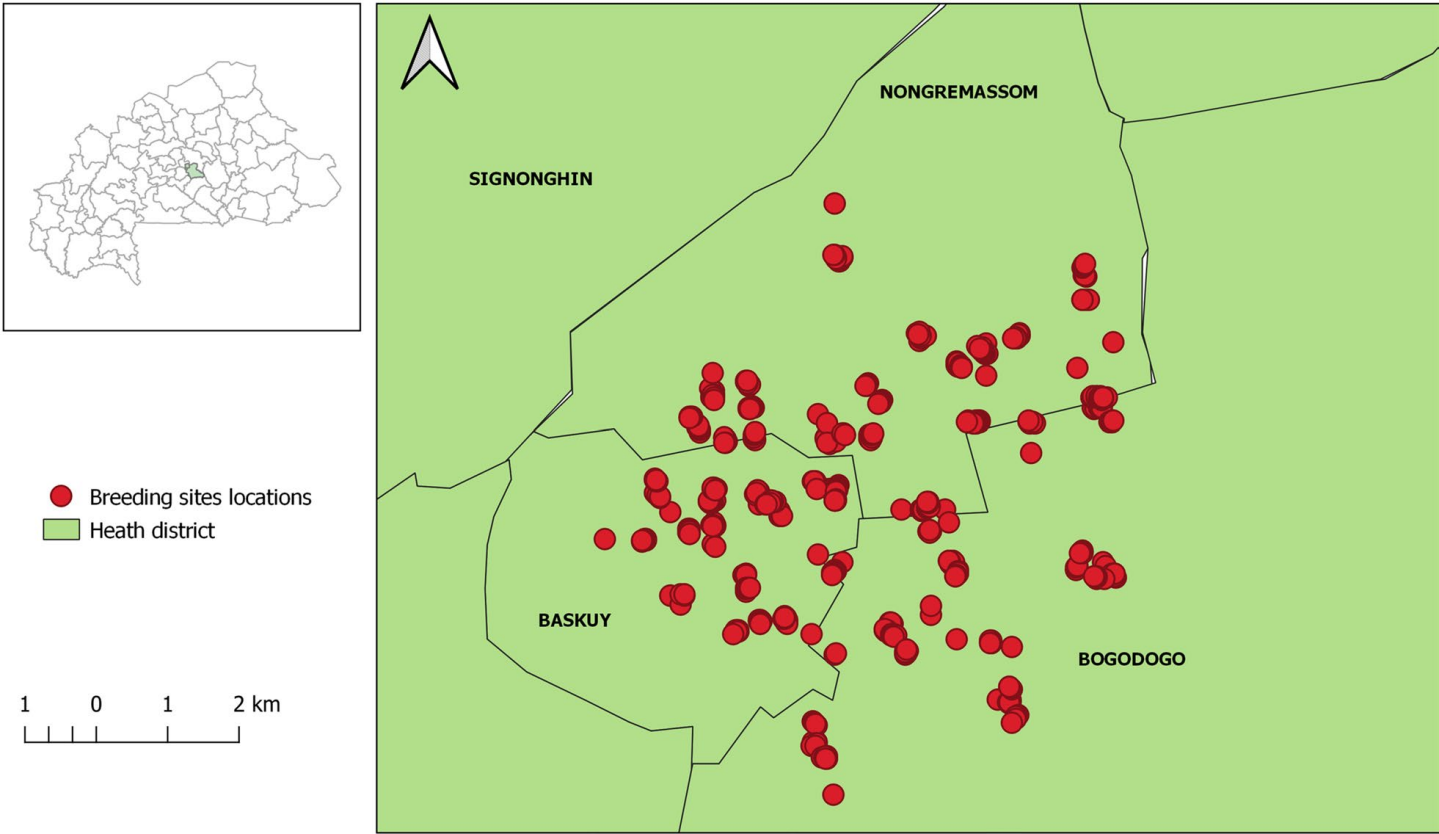
Map showing the locations visited in the three health districts. The red circles indicate the location of the households surveyed in the three health districts. The green area indicates the health districts (Baskuy, Bogodogo and Nongremassom)

The study was carried out during the rainy season from August to October 2018 in Ouagadougou City (12^o^22ʹN, 001°31ʹW), the capital city of Burkina Faso, located in the central region of the country. The daily average temperature during our study period ranged between a minimum of 23.7 °C and maximum 33.3 °C, with total rainfall of 435.3 mm (monthly average: 145.1 mm). The study involved three of the five central region health districts (HD) in Ouagadougou City: Baskuy (central part of city), Bogodogo (northern part) and Nongremassom (south-eastern part) (Fig. 1).

The three HD were selected based on the number of dengue cases reported during the outbreak of 2016–2017. In that outbreak, Bogodogo and Nongremassom recorded the highest numbers of dengue cases, whereas Baskuy recorded only a few cases. Both urban and peri-urban areas of Bogodogo and Nongremassom HD were included in the study, but only urban areas in Baskuy were included as this HD lacks peri-urban areas. In each HD, we visited approximately 200 households in ten neighbourhoods during the study period to collect samples from larval habitats both indoors and outdoors.

### Study design and field survey

In each HD, a house-to-house cross-sectional entomological survey was carried out to screen for water-holding containers both indoors and outdoors, with the aim to detect containers infested with immature stages of *Aedes* mosquitoes and to characterise the larval breeding sites identified. Each house was visited once. During the visit, all water-holding containers representing potential mosquito breeding sites, such as tyres, drums, cans and plastic or metal containers of a range of sizes and purposes, were carefully inspected for the presence of *Aedes* immature stages (larvae and pupae). Detailed characteristics of the breeding site containers were documented for all containers, including type and nature of container, diameter and size of container, water level and volume in container, physicochemical variables of water in container, sun exposure (sunny or shady) and surrounding vegetation. Containers found during the survey were grouped into six types that took the size of the container into account (Table 1): (i) tyres (unique category); (ii) medium-sized containers (buckets, cans and pots [BCP]); (iii) large containers (drums and barrels [DB]); (iv) small containers (SC); (v) water feeders (WF) for animal; and (vi) Others.

**Table 1:** Classification and definition of *Aedes aegypti* breeding site containers sampled in the three health districts

Types of breeding site containers	Container’s description
Tyres	Bicycle, motorcycle, car or any other discarded motor vehicle tyres
Buckets/cans/pots (BCP)	Discarded or unused bucket, can and pots (≥ 5 l and ≤ 50 l)
Drums/barrels (DB)	Plastic, metallic and ceramic containers for water storage use (> 50 l)
Water feeder for animals (WF)	Any type of container of any material used for the purpose of watering animals living in the yard
Small containers (SC)	Small discarded containers of any material (< 5 l)
Others	Diverse unclassified containers, such as basins, tree hole, used tables, used fridges, puddles of water, plastic bags

The physicochemical analysis of the water in each container breeding site was recorded directly in the field using a multi-parameter probe (multi-parameter COMBO de poche; Hanna Instruments, Woonsocket, RI, USA; product reference HI98129). The parameters measured included water temperature (°C), pH, electrical conductivity (EC; μS/cm) and total dissolved solids. For all water-holding containers we followed the WHO guidelines classification [36] and the type of material was also recorded. Additional characteristics of the breeding sites containers were documented for all containers, including location, sun exposure, container size, water level and volume.

All water-holding containers found were examined for mosquito larvae and pupae. All immature stages of mosquitoes were collected from the containers found to have mosquito larvae and pupae (positive containers) and transferred immediately into labelled plastic bottles filled with breeding site water. The bottles were brought to the laboratory at the University Joseph KI-ZERBO for sorting.

### Larvae and pupae processing in the laboratory

All third- and fourth-instar larvae (L3 and L4, respectively) from the positive containers were sorted by genus (*Aedes*, *Culex* or *Anopheles*) based on morphological criteria [37] and counted. First- and second-stage larvae were kept in an insectary and reared on dried fish food (Tetramin®; Tetra, Melle, Germany) to L3 or L4 before identification. Owing to the difficulty of identifying pupae, these were kept until the emergence of adults, when they were identified using the keys described by Huang [38] and Rueda [37]. After identification, mosquitoes were put into 1.5-ml Eppendorf tubes over silica gel, with the tubes labelled according to their species and type of breeding site container, and stored in the freezer at − 20 °C.

### Wing size measurement

The wings of male and female mosquitoes that emerged from collected larvae and pupae were measured as a proxy for adult body size. The wings of each mosquito were removed using a needle under a binocular microscope (Leica Microsystems GmbH, Wetzlar, Germany) before being placed on a micrometric slide (with 0.1 mm gradation) and photographed using a camera attached to the microscope. Wing measurements were made from the image files using Image J 1.42 software (http://rsbweb.nih.gov/ij/). Wings were measured from the alula to the apical margin, excluding the fringe [39]. We used the mean length of the two wings raised to the cube as an index of mosquito body size [40].

### Data analysis

The level of infestation was estimated using traditional *Stegomyia* indices, including the Breteau index (BI, the number of positive containers per 100 surveyed houses), house index (HI, the percentage of houses infested) and container index (CI, percentage of positive containers) [36, 41]. The WHO established estimated thresholds of these indices for dengue and yellow fever transmission [15]. The container preferences of *Ae. aegypti* for breeding were assessed by calculating the breeding preference ratio (BPR), defined as the percentage of a specific container with *Ae. aegypti* immatures divided by the total density of that specific container [42]. The BPR measures the level of preference of *Ae. aegypti* females for available container types.

All data analyses were performed using R version 3.6.3 software ® Foundation for Statistical Computing, Vienna, Austria). The abundance of immatures and pupae productivity were estimated for each type of water-holding container. Proportions were compared using the Chi-square test. Pupal productivity was assessed as the number of pupae produced at a given type of breeding site divided by the total number of pupae produced at all breeding sites, multiplied by 100 [36].

Generalised linear mixed models (GLMMs) with a negative binomial link function using the R package “glmmTMB” [43] were fitted to *Ae. aegypti* larval and pupal counts separately, with container physicochemical characteristics, rainfall and temperature as covariates. Specifically, for larval and pupal abundance, we used container type, HD, month, pH and EC of water, respectively, water level, the cumulative pluviometry of last 10 days and container diameter as covariates. We included as interaction terms HD × container and HD × month, and to account for variation arising from the sampling design we included date of collection and house identifier as random effects. For pupae, variables such as month and pH were used. From these full models, the minimal model was selected using a stepwise backward procedure based on the lowest Akaike information criterion (AIC) values by removing factors with the highest* P*-value in the model. If removal of a variable resulted in a change in the AIC value of > 2, the resultant model was still parsimonious and based on residual diagnostics in DHARMa [44], the simplified model was kept. This procedure was repeated until the removal of variables no longer improved the model.

GLMMs with normal linked function were run for wing length to the power of 3 as a proxy for *Ae. aegypti* mosquito (male and female) body size. *Aedes aegypti* wing length was regressed against locality, collection month, container type, breeding site location (shady or sunny), mosquito gender (for body size), physicochemical variables (temperature, EC and pH of water) and climate factors (temperature, rainfall). HD × container and HD × month were included as interaction terms, and date of collection and house identifier were added as random effects. Minimal model selection proceeded as above for abundance modelling. A significance level of *P* = 0.05 was set for all statistical analyses.

### Ethical considerations

The study was approved by the Ethical Research Committee of the Ministry of Health (No. 2017-8-0126 of 02/08/2017). Signed informed consent was obtained from all householders included in the study before the field collection was started.

## Results

### *Stegomyia* indices

From August to October 2018 a total of 351 houses in three HD were visited, of which 54.6% were found to harbour at least one container positive for *Ae. aegypti* larvae and/or pupae (Table 2). A total of 1061 water-holding containers were inspected, of which 760 (71.6%) were positive for *Ae. aegypti* larvae or pupae (Table 2). The *Stegomyia* indices of the three HD were all above the WHO threshold values for yellow fever, which are HI, BI and CI values of 35%, 50% and 20%, respectively [45]. The lowest HI and BI values were 51.4% and 108.2%, respectively, recorded in Bogodogo, and the lowest CI value was 67.8%, recorded in Nongremassom. All recorded *Stegomyia* indices were higher than the WHO threshold values, suggesting a high level of *Ae aegypti* infestation. Both house positivity (*χ*^2^ = 18.29, *P* < 0.001) and container positivity (*χ*^2^ = 6.83, *P* = 0.033) varied significantly between localities.

**Table 2:** Number of houses, containers and entomological indices for each health district

Health districts	Visited houses (*n*)	Positive houses (*n*)^a^	Prospected containers (*n*)	Positive containers (*n*)	Entomological indices^b^		
					HI (%)	CI (%)	BI
Baskuy	194	111	356	272	57.2	76.4	140.2
Bogodogo	220	113	336	238	51.4	70.8	108.2
Nongremassom	229	127	369	250	55.5	67.8	109.2
Total	643	351	1061	760	54.6	71.6	118.2

^a^Houses where containers infested with immature stages of *Aedes* mosquitoes were found (‘positive’)

^b^HI, House index: the proportion of houses with at least 1 *Aedes aegypti*-positive container. CI, Container index: the proportion of containers with at least 1 *Ae. aegypti* immature stage. BI, Breteau index: the number of *Ae. aegypti*-positive containers for 100 houses

### Breeding site abundance and distribution

The prevalence of water-holding containers in each HD is shown in Table S1. There were no statistically significant differences between the proportions of the different types of potential breeding sites across the HD (*χ*^2^ = 12.13, *P* = 0.27), with tyres being the most common potential container breeding site, followed by DB container types and then BCP container types. Of 760 positive containers, tyres were the most prevalent breeding containers in the three HD, followed by BCP in Baskuy and Bogodogo and by DB in Nongremassom. The prevalence of tyres was not significantly different between HD (*χ*^2^ = 2.68, *P* = 0.26).

Tyres also had the highest positivity ratio in each of the three HD (Additional file 1: Table S1), with no significant difference between the positivity ratio of tyres among HD (*χ*^2^ = 0.13, *P* = 0.94). When the positivity ratio among containers was compared based on container material, plastic containers were the second-most positive containers (after tyres, rubber) in Baskuy and Bogodogo; in Nongremassom, ceramic containers were the second-most positive container type.

### Preferred oviposition sites of *Ae. aegypti*

Tyres were found to be the most preferred container type, with the BPR ranging from 1.1 to 1.26 (Additional file 1: Table S1). The BPR for other container types varied according to HD, with water storage containers, WF and SC being the second-most preferred containers in Baskuy, Bogodogo and Nongremassom, respectively (Additional file 1: Table S1).

### *L*arval productivity and abundance of* Ae. aegypti*

**Fig. 2:**
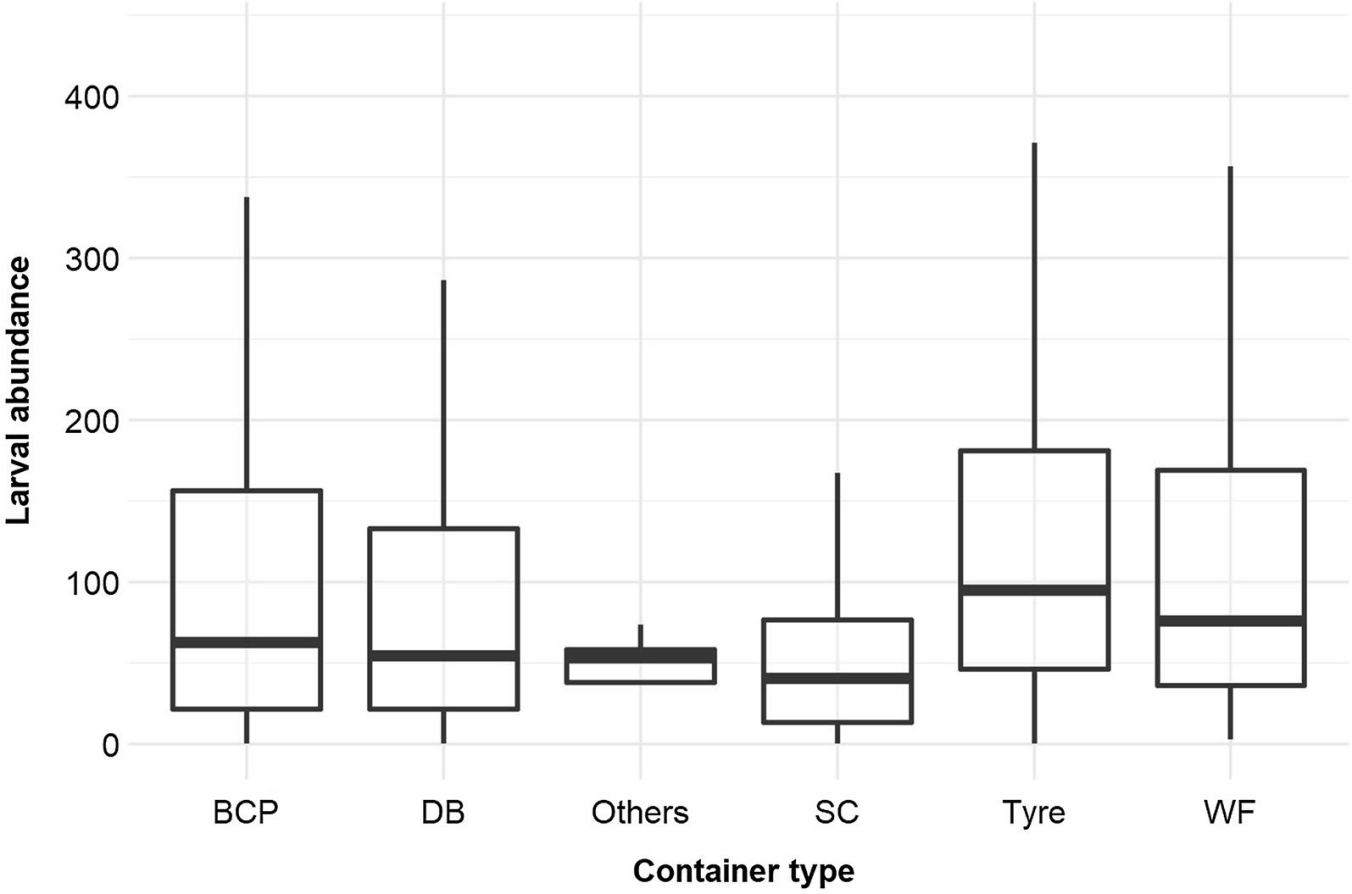
Box-plot analysis of *Aedes aegypti* larval abundance according to breeding container type. BCP, Buckets/cans/pots (medium-sized containers); DB, drum/barrel (large-sized containers); SC, small containers; WF, water feeders

Tyres, medium-sized containers (BCP) and large-sized containers (DB) accounted for > 80% of the total larvae in each HD. In all three HD, the highest larvae production occurred in tyres, accounting for 37.3% of larvae production in Baskuy, 50.8% in Bogodogo and 40.1% in Nongremassom. The second-most important container type for larval production in Bogodogo (18.0%) and Nongremassom (32.1%) was DB, while in Baskuy, medium-sized containers (BCP) ranked second (36.6%) (Additional file 1: Table S1). The cumulative contribution to larval production of these containers was 86.2%, 86.3% and 83.9%, respectively, for Baskuy, Bogodogo, and Nongremassom. In Bogodogo, the larvae recorded in tyres accounted for > 50% of all larvae detected (50.8%). Significant factors included in the minimal abundance GLMM for larval abundance were HD, container type, collection month, cumulative rainfall (over 10 days), container diameter, EC, pH and the interaction of HD × container type, suggesting local-specific differences in usage (Table 3). Mean larval abundance varied between container types, with tyres supporting the highest number of *Ae. aegypti* (Fig. 2; Additional file 2: Figure S1).

**Table 3:** Generalised linear mixed model of *Aedes aegypti* larval abundance showing predictors, beta estimates of effect size, test statistic (*Z*-value) and associated probability for the minimal model

Predictors^a,b^	Estimate	Standard error	*Z*-value	Pr( >|z|)
**(Intercept)**	**8.09**	**0.77**	**10.62**	**< 0.001**
*Health.District [Baskuy]*				
Bogodogo	− 0.331	0.246	− 1.35	0.18
** Nongremassom**	**− 0.82**	**0.26**	**− 3.18**	**0.001**
*Container [BCP]*				
** DB**	− **0.77**	**0.24**	− **3.211**	**0.001**
** SC**	− **0.79**	**0.254**	− **3.16**	**0.002**
** Tyre**	− **0.51**	**0.21**	− **2.46**	**0.014**
** WF**	− **0.79**	**0.35**	− **2.26**	**0.024**
** Others**	− **1.42**	**0.81**	− **1.76**	**0.08**
*Month [August]*				
** September**	− **0.42**	**0.14**	− **3.00**	**0.003**
** October**	− **0.67**	**0.16**	− **4.24**	** < 0.001**
**Container diameter (cm)**	**0.01**	**0.00**	**3.37**	** < 0.001**
**Water level (cm**)	**0.03**	**0.01**	**2.84**	**0.004**
**Electrical conductivity (μS/cm)**	**0.22**	**0.08**	**2.62**	**0.008**
**pH**	− **0.46**	**0.10**	− **4.42**	** < 0.001**
*Health.District [Baskuy]:Container[BCP]*				
Bogodogo: DB	0.50	0.37	1.35	0.18
** Nongremassom: DB**	**1.54**	**0.36**	**4.24**	** < 0.001**
Bogodogo: Others	0.08	1.40	0.06	0.951
Nongremassom: Others	− 68.97	137.64	− 0.50	0.62
Bogodogo: SC	0.04	0.40	0.09	0.925
** Nongremassom: SC**	**0.77**	**0.39**	**2.00**	**0.045**
Bogodogo: Tyre	0.45	0.30	1.49	0.135
** Nongremassom: Tyre**	**0.90**	**0.31**	**2.88**	**0.003**
Bogodogo: WF	0.71	0.51	1.41	0.159
** Nongremassom: WF**	**1.06**	**0.51**	**2.06**	**0.04**

Non-significant terms not included in the model were: vegetation presence/absence, temperature, relative humidity, container height, container usefulness, container material, container utility, container size, water volume, container location, number of persons, health district × month, temperature × location

^a^Reference categories are shown in square brackets

^b^Significant predictors are highlighted in bold font and non-significant predictors are listed immediately thereunder

The pH of the water of the container breeding sites varied from 4.72 to 9.33 and was found to be significantly negatively associated with larval abundance (Table 3), i.e. the lower the pH, the higher the larval abundance. Larval abundance decreased significantly across months, with the highest larval abundance being recorded in August (the wettest month) (Table 3; Additional file 2: Figure S2).

### Pupal productivity and abundance of *Ae. aegypti*

**Fig. 3:**
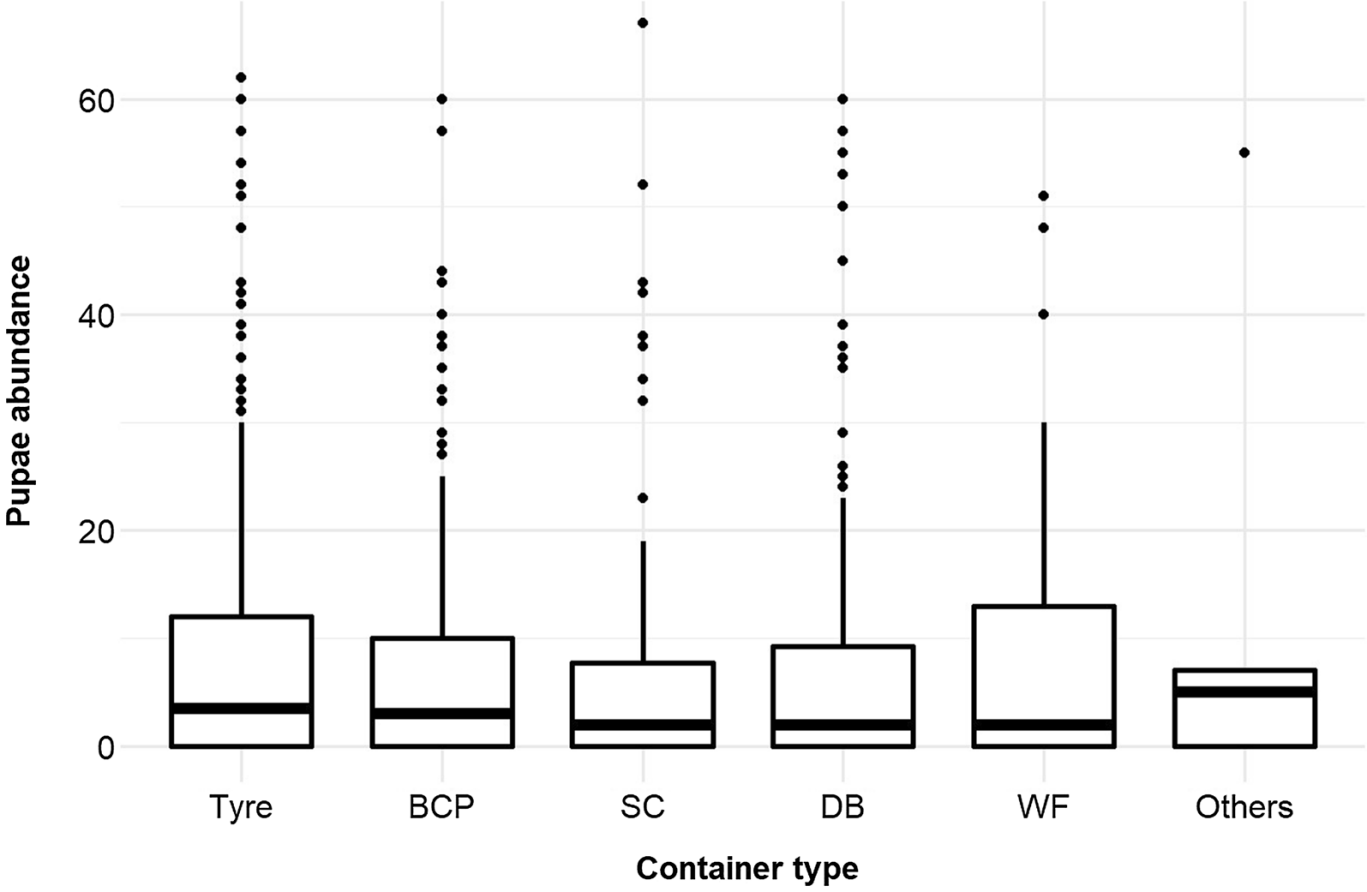
Box-plot analysis of pupal abundance of *Ae. aegypti* according to breeding container type

In Baskuy and Bogodogo, tyres were the most productive pupal containers, with 33.8% and 56.7% of tyres found to be positive, respectively; in Nongremassom, large-sized containers (DB) were the most productive pupal containers (36.60%), followed by tyres (30.4%) and medium-sized containers (BCP; 18.1%). In Baskuy, the second- and third-most productive pupal containers (following tyres) were SC (23.8%) and BCP (20.4%); in Bogodogo, the second-most productive containers for pupae were BCP (16.5%) and DB (16.2%) (Fig. 3; Additional file 1: Table S1).

The pupal GLMM showed a significant difference in pupal abundance between months, with a decline in September and October (Table 4; Additional file 2: Figure S3). Unlike the association between larval abundance and breeding site types, there was no difference in pupal abundance between breeding site types, although the significant interaction location × site terms suggested some heterogeneity in usage among HD (Table 4). Similar to its negative effect on larval abundance, pH was also negatively associated with pupal abundance (Table 4).

**Table 4:** Generalised linear mixed model of *Ae. aegypti* pupal abundance showing predictors, beta estimates of effect size, test statistic (*Z*-value) and associated probability for the minimal model

Predictors^a,b^	Estimate	Standard error	*z*-value	Pr( >|z|)
**(Intercept)**	**5.08**	**1.10**	**4.63**	** < 0.001**
*Health district [Baskuy]*				
Bogodogo	0.06	0.34	0.18	0.86
Nongremassom	0.52	0.36	1.45	0.146
*Container [BCP]*				
DB	0.05	0.35	0.13	0.895
SC	0.70	0.36	1.95	0.051
Tyre	0.01	0.29	0.03	0.979
WF	0.30	0.53	0.54	0.590
Others	1.59	1.22	1.300	0.193
*Month [August]*				
** September**	− **0.47**	**0.18**	− **2.57**	**0.010**
** October**	− **0.63**	**0.21**	− **3.04**	**0.002**
**pH**	− **0.40**	**0.15**	− **2.57**	**0.010**
H*ealth district [Baskuy] × container [BCP]*				
Bogodogo: DB	0.25	0.54	0.46	0.643
Nongremassom: DB	0.60	0.53	1.13	0.260
Bogodogo: Others	− 1.75	2.13	− 0.82	0.410
Nongremassom: Others	− 0.22	988.5	− 0.00	0.998
** Bogodogo: SC**	− **1.39**	**0.61**	− **2.28**	**0.022**
** Nongremassom: SC**	− **1.51**	**0.575**	− **2.63**	**0.009**
Bogodogo: Tyre	0.46	0.44	1.04	0.296
Nongremassom: Tyre	− 0.22	0.45	− 0.49	0.624
Bogodogo: WF	− 0.91	0.75	− 1.22	0.224
Nongremassom: WF	− 0.30	0.76	− 0.38	0.705

Non-significant terms were, vegetation presence/absence, temperature, relative humidity, container height, container utility, container material, container size, water volume, container location, number of persons, health district × month, temperature × location

^a^Reference categories are shown in square brackets []

^b^Significant predictors are highlighted in bold text, and non-significant terms not included in the model are listed thereunder

### Adult body size and associated factors

The GLMM of *Ae. aegypti* body size and types of water-holding containers revealed that the size of adult mosquitoes emerging from containers was significantly influenced by the type and characteristics of the container. Specifically, mosquitoes from tyres and large containers (DB) had smaller body sizes. Container characteristics such as diameter were positively associated with adult body size (Table 5).

**Table 5:** Generalised linear mixed model of *Ae. aegypti* mosquito body size showing predictors, beta estimates of effect size, test statistic (*t*-value) and associated probability for the minimal model

Predictors^a,b^	Estimate	Standard error	*Z*-value	Pr( >|t|)
**(Intercept)**	**29.91**	**2.85**	**10.5**	** < 0.001**
*Health district [Baskuy]*				
Bogodogo	1.55	1.22	1.27	0.204
Nongremassom	0.64	1.06	0.61	0.543
*Month [August]*				
September	0.69	0.79	0.88	0.380
** October**	**7.57**	**0.92**	**8.20**	** < 0.001**
**Ten days of rainfall**	**0.032**	**0.011**	**3.01**	**0.003**
Container [BCP]				
** Drum**	− **4.82**	**0.81**	− **5.93**	** < 0.001**
** Tyre**	− **5.41**	**0.82**	− **6.61**	** < 0.001**
**Temperature**	− **0.41**	**0.09**	− **4.61**	** < 0.001**
**Container diameter (cm)**	**0.06**	**0.01**	**8.76**	** < 0.001**
**pH**	− **0.77**	**0.18**	− **4.41**	** < 0.001**
**Electrical conductivity**	**1.68**	**0.55**	**3.04**	**0.002**
**Location in sun**	− **1.51**	**0.61**	− **2.46**	**0.014**
***Gender [Female]*** **Male**	− **6.347**	**0.30**	− **21.09**	** < 0.001**
*Health district[Baskuy] × month[August]*				
** Bogodogo: October**	− **11.31**	**1.26**	**− 8.94**	** < 0.001**
** Nongremassom: October**	− **8.94**	**2.02**	− **4.42**	** < 0.001**
Bogodogo: September	− 1.99	1.07	− 1.86	0.064
Nongremassom: September	− 1.27	1.11	− 1.15	0.252
*Health district [Baskuy] × Container [BCP]*				
Bogodogo: Drum	− 1.00	1.15	− 0.87	0.383
** Nongremassom: Drum**	**3.29**	**1.13**	**2.92**	**0.004**
Bogodogo: Tyre	2.06	1.06	1.95	0.051
Nongremassom: Tyre	1.33	1.02	1.31	0.192

Non-significant terms were: vegetation presence/absence, temperature, relative humidity, container height, container usefulness, container material, container size, water volume, container location, number of persons, health district × month, temperature × location

^a^Reference categories are shown in square brackets []

^b^Significant predictors are highlighted in bold text, and non-significant terms not included in the model are listed thereunder

**Fig. 4:**
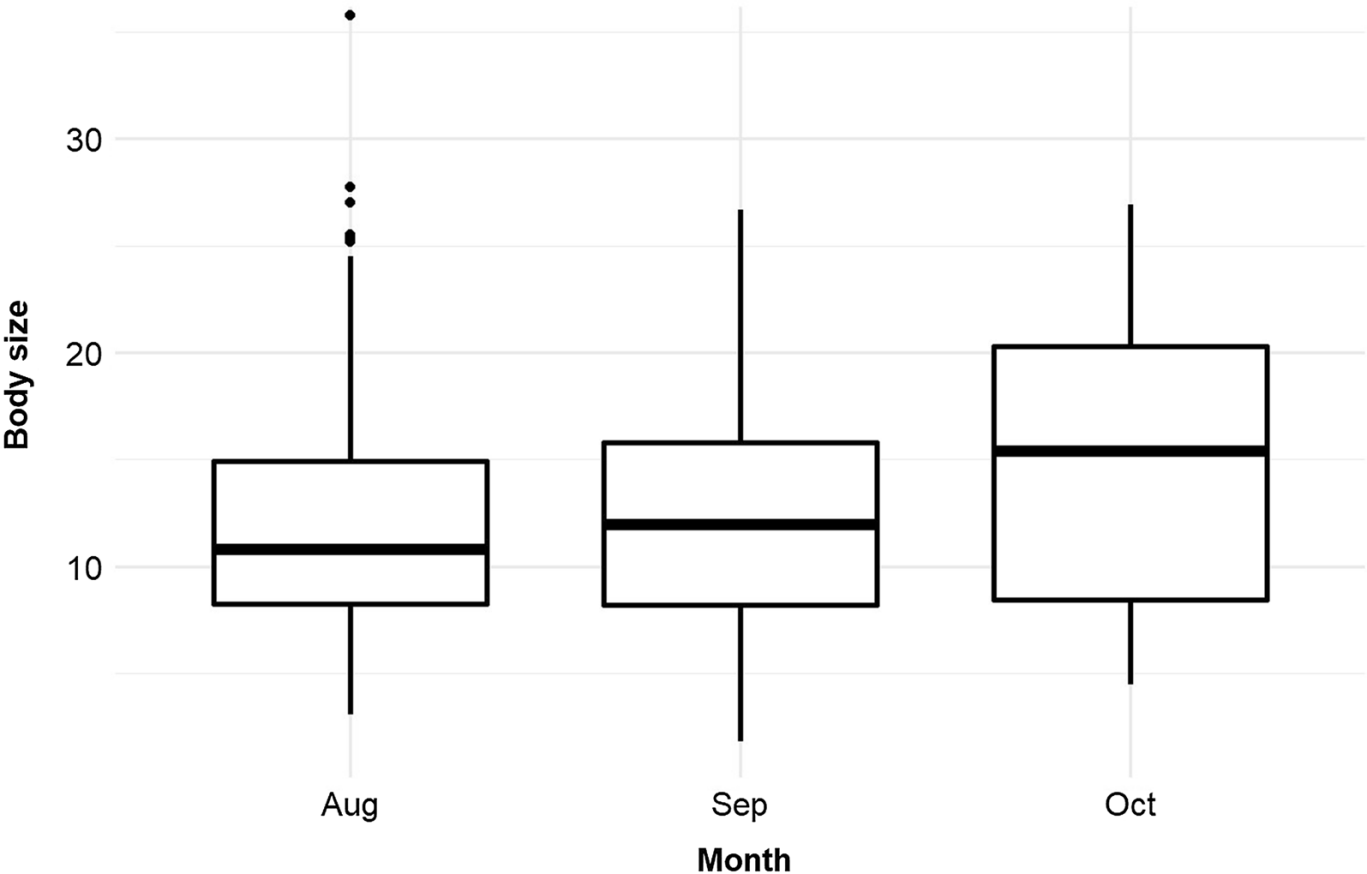
Box-plot of *Ae. aegypti* mosquito adult body size according to collection period. Aug, August); Sep, September; Oct, October

Body size also varied with month of collection (Fig. 4), with larger mosquitoes found during October and a significant interaction term indicating inconsistency between container types among HD (Table 4; Fig. 4). Significant negative associations were found between mosquito body size and water temperature, exposure to sunshine and pH. Water conductivity, 10 days of cumulative rainfall and container diameter were positively associated with mosquito body size (Table 5). Female body size was significantly larger than that of males (Table 5).

## Discussion

Arboviral infections are predicted to increase worldwide, driven mainly by anthropogenic changes to the environment that include, among others, climate change, urbanisation and changes in land uses [46]. The African continent is particularly at increased risk, with a projected increase in arbovirus threat and a decrease in malaria incidence [47]. Anthropogenic changes create favourable ecological niches for the proliferation of *Ae. aegypti*. A better understanding of how breeding site characteristics influence the productivity and abundance of immature mosquitoes is important to predict the success of policies that target larval source management of *Ae. aegypti*. The present study combined estimation of traditional *Stegomyia* indices with assessment of how container type, physicochemical characteristics of the water in the container and climate affect larval and pupal abundance, as well as mosquito body size. Our findings show that *Stegomyia* indices of the three HD in Ouagadougou City are all above the WHO thresholds. Our results also show that in all HD, tyres, DB and BCP were not only the most frequent potential larval breeding sites, but also had the highest proportions of positivity and productivity. Breeding container type also affected the mosquito body size width.

### High entomological risk of arbovirus transmission

High *Stegomyia* indices, i.e. those exceeding WHO thresholds [48], were recorded in all three HD included in the present study, suggesting a high level of infestation by *Ae. aegypti* and, thereby, a higher risk of transmission of arboviruses in general and dengue in particular, with cases reported on a regular basis. Indeed, our study period corresponded with the peak of dengue cases in Ouagadougou in 2018 [49]. *Stegomyia* indices exceeding WHO thresholds were reported in and around Ouagadougou during the two consecutive and officially recognised outbreaks that occurred in 2016 and 2017 [8, 9, 17]; these outbreaks resulted in 2600 reported cases/21 deaths and 14,455 reported cases/29 deaths, respectively. *Stegomyia* indices were higher in 2018 although disease burden remained substantial, with 4386 dengue cases and 25 deaths. Two factors might contribute to this lack of correspondence. The first is a general lack of evidence of any association between *Stegomyia* indices and dengue outbreaks [50]. The second is inconsistency in the methods used for declaring outbreaks [51]. Even though not linked to outbreaks each year, these first records from Burkina Faso that link high indices to high disease occurrence are useful for comparative purposes on the level of infestation by *Aedes* mosquitoes and highlight the need for continued *Aedes* surveillance in all at-risk areas in the region.

### *Ae. aegypti* breeding site profiles

*Aedes aegypti* immature stages were found colonising different type of water-holding containers, with used tyres being the most common, followed by large-sized containers (DB) and medium-sized containers (BCP). A study in and near Ouagadougou in 2016 and 2017 recorded similar results: in urban and semi-urban localities tyres were found to be among the most common and most productive breeding sites while in the rural sites, DB were more important type [17]. In another previous study, which included other localities in Ouagadougou, the importance of discarded containers, mainly tyres, was also highlighted [25]. DB used for water storage were the second most frequent breeding sites in Bogodogo and Nongremassom and the third in Baskuy. Ridde et al. [52] found in some neighbourhoods of Ouagadougou that DB, when used as water storage containers, were among *Ae. aegypti* most frequent breeding sites. The prevalence of DB could be associated with the absence or inaccessibility of piped water supplies or irregular functioning of these piped water systems in some areas [42, 53, 54].

The results of the present study are also consistent with findings from other African countries (Côte d’Ivoire, Kenya, Mozambique and Ethiopia) where tyres were found to be the most predominant water-holding containers and also to have the highest positivity ratio and the highest abundance of immatures [13, 53, 55, 56]. When we classified the breeding sites according to the container material, rubber (i.e. tyres), plastic and ceramic containers had the highest numbers and positivity rates, consistent with results from previous studies in Ouagadougou [17, 52] and in Zanzibar [54].

These results suggest that larval site reduction may best be approached by targeting the most productive containers, which in turn may reduce arbovirus risk in Ouagadougou. This approach should involve communities, tyre retailers and the municipalities.

### Factors affecting *Ae. aegypti* immature abundance

*Aedes aegypti* immature abundance was affected by the physical parameters of the containers, chemical parameters of the water within the containers and environmental parameters external to containers. Previous studies have also detected an impact of the physicochemical parameters of the water in containers, such as salinity, EC, dissolved oxygen and pH on *Aedes* immature stages or productivity [57, 58]. The results of our study suggest a negative correlation between *Ae. aegypti* immature abundance and water pH (range: 4.72–9.33 in *Ae. aegypti*-positive containers), in contrast with results from a study in India which found a positive correlation between larval abundance and pH (range: 6.72–7.63) [57]. Although it is difficult to translate these multiple effects and interactions of different factors into vector control recommendations, it is important to note that factors affecting larval density are different from those affecting pupal density and may explain why larval abundance does not correlate well with adult abundance [17]. Month and pH were the only factors affecting both larval and pupal density. Factors affecting abundance and even abundance itself may also impact mosquito body size, which may have epidemiological implications for immature’s life carry-over effects on *Ae. aegypti* competence for arbovirus transmission [29, 30].

### Impact of breeding site characteristics on *Ae. aegypti* adult body size

Mosquito body size is generally associated with environmental factors [59], and we found that the type of breeding site affected *Ae. aegypti* mosquito body size. Indeed, we observed that adults emerging from medium-sized containers (BCP) had a larger average body size than those emerging from tyres and large-sized containers (DB). Most drums are used for clean water storage and contain relatively little detritus; consequently, this water contains relatively lower nutrient levels for mosquito larvae. The higher larval abundance observed in tyres may account for the smaller body size of the adult mosquitoes that emerge from this type of container [60, 61], as larval competition is a known important determinant of adult body size for *Aedes* and other culicid species [60]. We found that temperature had a negative association with mosquito body size, consistent with results reported in previous studies [30, 62, 63] which shows that body size of mosquitoes decreases when the temperature increases. The average body size of the *Aedes* mosquito in October was larger, and the abundance of immature stages lower, than in September and August; this result is consistent with mosquito body size being density-dependant in their natural environment due to competition for food and space.

While the effects of body size are likely to be manifold and complex, *Aedes* body size appears to affect blood-feeding patterns in the laboratory, with small-sized individuals more likely to take multiple blood meals and thus potentially having a direct impact on the probability of transmitting arboviruses [64, 65].

## Conclusion

The results of the present study provide insight into the most prevalent and productive potential breeding containers and the consequences for the adult *Ae. aegypti* that emerge from them. We found that factors such as locality, month and container types affected immature abundance as well as adult body size. Although the properties of a container and the physicochemical factors of the water it contains undoubtedly influence the proliferation of a local mosquito population as well as adult mosquito body size, only the EC and pH of the water were found to affect both immature abundance and mosquito body size. There is no clear justification of targeting specific breeding sites based on their physicochemical characteristics as these factors seem to have limited effect on immature abundance and body size. However, tyres, which were found to be the most productive breeding site container of immature stages of *Ae. aegypti*, might be given priority in a strategy aimed at reducing the number of potential breeding containers.

## Supplementary Information

**Additional file 1: Table S1.** Breeding site container classification, prevalence positivity, productivity and *Aedes aegypti *mosquito breeding site preference ratio (BPR) according to container type and health district.

**Additional file 2: Figure S1.** Classification of breeding sites according to the container material. The percentage show the proportion of each type of material according to the health district. Values in brackets indicate the number of breeding sites in each district. **Fgure S2.** Graph of* Aedes aegypti* mosquito larval abundance according to collection period. **Figure S3.** Graph of pupal abundance according to collection period. Aug, August; Sep, September; Oct, October.

## Data Availability

The authors confirm that all data underlying the findings are fully available without restriction. All relevant data are within the paper and its Supporting Information files.

## Abbreviations

BCP: Buckets, cans and pots
BI: Breteau index
BRP: Breeding site preference ratio
CI: Container index
DB: Drums and barrels
GLMM: Generalised linear mixed model
HD: Health district
HI: House index
SC: Small containers
WF: Water feeders for animals
