# Supplementary material for: Impact of physicochemical parameters of Aedes aegypti breeding habitats on mosquito productivity and the size of emerged adult mosquitoes in Ouagadougou City, Burkina Faso
Source: Parasit Vectors. 2022 Dec 20;15:478. doi: 10.1186/s13071-022-05558-3 (PMC9768987; doi:10.1186/s13071-022-05558-3)
Supplement: Supplementary file 1 — Additional file 1: Table S1. Breeding site container classification, prevalence positivity, productivity and Aedes aegypti mosquito breeding site preference ratio (BPR) according to container type and health district. [file 13071_2022_5558_MOESM1_ESM.docx]

**Table S1:** Breeding site container classification, positivity, productivity and *Aedes aegypti* mosquito breeding site preference ratio (BPR) according to container type and Health district

| **Breeding site containers** | **Number (%)** | **Positive containers (%)** | **Positivity % by container type** | **Larval production** | **Pupal production** | **Larval productivity (%) by container type** | **Pupal productivity (%) by container type** | **Breeding Preference Ratio (BPR**^*^**)** |
| --- | --- | --- | --- | --- | --- | --- | --- | --- |
| **Health District of BASKUY** | | | | | | | | |
| Tires | 117 (32.80) | 98 (36.00) | 83.80 | 16,646 | 971 | 37.30 | 33.80 | 1.1 |
| Bucket/Can/Pot (BCP) | 100 (28.10) | 76 (27.90) | 76.00 | 16,335 | 586 | 36.60 | 20.40 | 0.99 |
| Drums/Barrels (DB) | 54 (15.20) | 42 (15.40) | 77.80 | 5,491 | 362 | 12.30 | 12.60 | 1.02 |
| Small Containers (SC) | 57 (16.00) | 40 (14.70) | 270.20 | 4,642 | 685 | 10.40 | 23.80 | 0.92 |
| Water Feeders (WF) | 23 (6.50) | 14 (5.20) | 60.90 | 1,481 | 217 | 3.30 | 7.60 | 0.8 |
| Others | 5 (1.40) | 2 (0.70) | 40.00 | 96 | 55 | 0.20 | 1.90 | 0.53 |
| **Total** | **356** | **272** | **-** | **44,691** | **2,876** | **100** | **100** | **-** |
| **Health District of BOGODOGO** | | | | | | | | |
| Tires | 121 (36.00) | 102 (42.90) | 84.30 | 19,455 | 1,306 | 50.80 | 56.70 | 1.19 |
| Bucket/Can/Pot (BCP) | 75 (22.30) | 49 (20.60) | 65.30 | 6,685 | 380 | 17.50 | 16.50 | 0.92 |
| Drums/Barrels (DB) | 78 (23.20) | 42 (17.70) | 53.90 | 6,894 | 372 | 18.00 | 16.20 | 0.76 |
| Small Containers (SC) | 37 (11.00) | 26 (10.90) | 70.30 | 4,642 | 102 | 6.70 | 4.40 | 0.99 |
| Water Feeders (WF) | 21 (6.30) | 16 (6.70) | 76.20 | 1,481 | 132 | 6.70 | 5.70 | 1.08 |
| Others | 4 (1.20) | 3 (1.20) | 75.00 | 133 | 12 | 0.30 | 0.50 | 1.06 |
| **Total** | **336** | **238** | **-** | **38,276** | **2,304** | **100** | **100** | **-** |
| **Health District of NONGREMASSOM** | | | | | | | | |
| Tires | 110 (29.80) | 94 (37.60) | 85.50 | 17,493 | 1,071 | 40.10 | 30.40 | 1.26 |
| Bucket/Can/Pot (BCP) | 74 (20.10) | 43 (17.20) | 58.10 | 5,114 | 635 | 11.70 | 18.10 | 0.86 |
| Drums/Barrels (DB) | 94 (25.50) | 56 (22.4) | 59.60 | 14,020 | 1,285 | 32.10 | 36.60 | 0.88 |
| Small Containers (SC) | 65 (17.60) | 43 (17.20) | 66.50 | 4,299 | 281 | 9.90 | 8.00 | 0.98 |
| Water Feeders (WF) | 24 (6.50) | 14 (5.60) | 58.30 | 2,700 | 243 | 6.20 | 6.90 | 0.86 |
| Others | 2 (0.50) | 00 (0.0) | 0 | 0 | 0 | 0.0 | 0.0 | 0 |
| **Total** | **369** | **250** | **-** | **43,626** | **3,516** | **100** | **100** | **-** |
| **Grand Total** | **1,061** | **760** | **-** | **126,593** | **8,696** | **-** | **-** | **-** |
