# Supplementary material for: Impact of physicochemical parameters of Aedes aegypti breeding habitats on mosquito productivity and the size of emerged adult mosquitoes in Ouagadougou City, Burkina Faso
Source: Parasit Vectors. 2022 Dec 20;15:478. doi: 10.1186/s13071-022-05558-3 (PMC9768987; doi:10.1186/s13071-022-05558-3)
Supplement: Supplementary file 2 — Additional file 2: Figure S1. Classification of breeding sites according to the container material. The percentage show the proportion of each type of material according to the health district. Values in brackets indicate the number of breeding sites in each district. Fgure S2. Graph of Aedes aegypti mosquito larval abundance according to collection period. Figure S3. Graph of pupal abundance according to collection period. Aug, August; Sep, September; Oct, October. [file 13071_2022_5558_MOESM2_ESM.pptx]

## Slide 1
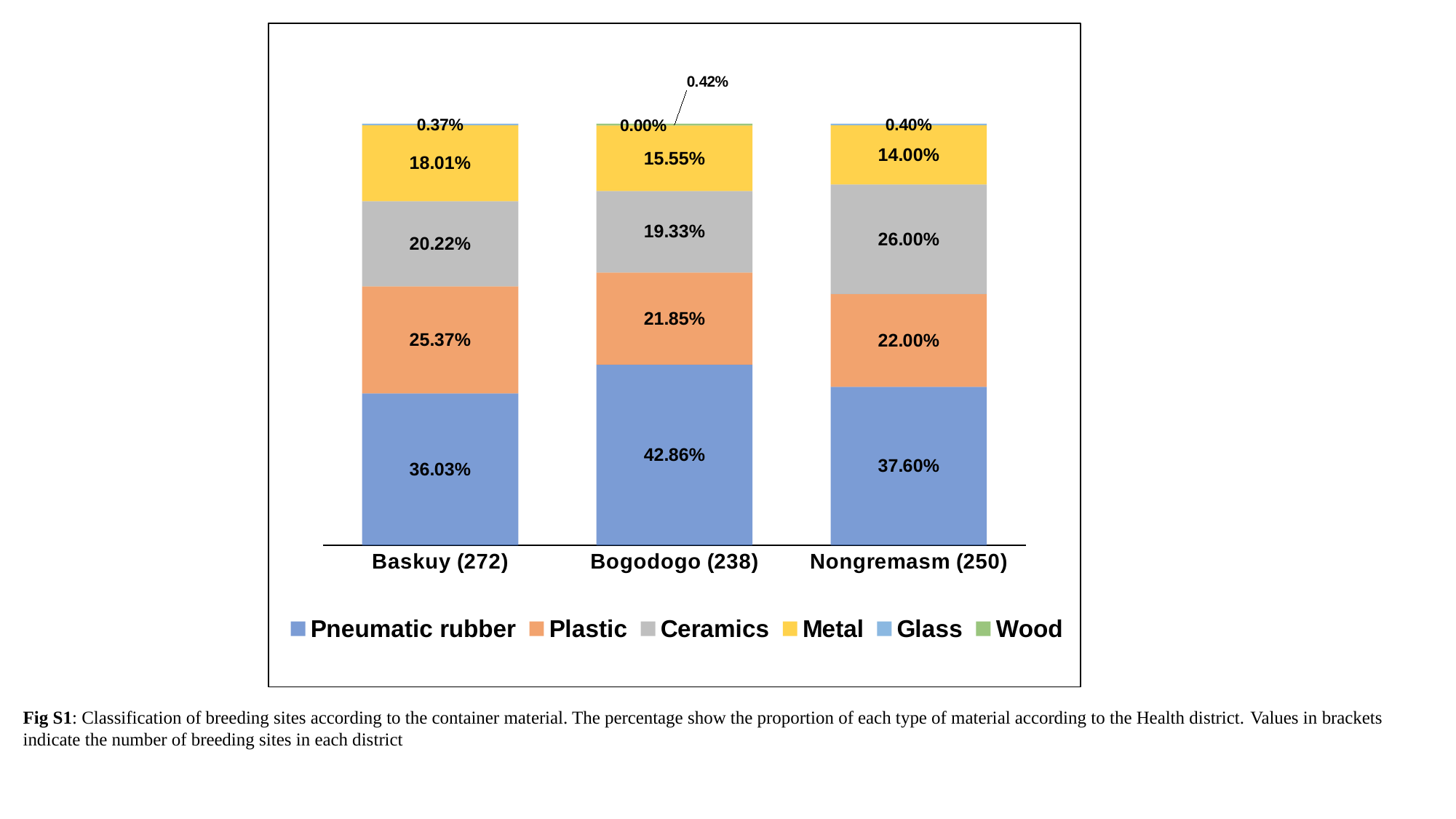

### Chart
| Category | Pneumatic rubber | Plastic | Ceramics | Metal | Glass | Wood |
|---|---|---|---|---|---|---|
| Baskuy (272) | 0.3602941176470588 | 0.2536764705882353 | 0.20220588235294118 | 0.1801470588235294 | 0.003676470588235294 | 0.0 |
| Bogodogo (238) | 0.42857142857142855 | 0.2184873949579832 | 0.19327731092436976 | 0.15546218487394958 | 0.0 | 0.004201680672268907 |
| Nongremasm (250) | 0.376 | 0.22 | 0.26 | 0.14 | 0.004 | 0.0 |Fig S1: Classification of breeding sites according to the container material. The percentage show the proportion of each type of material according to the Health district. Values in brackets indicate the number of breeding sites in each district

## Slide 2
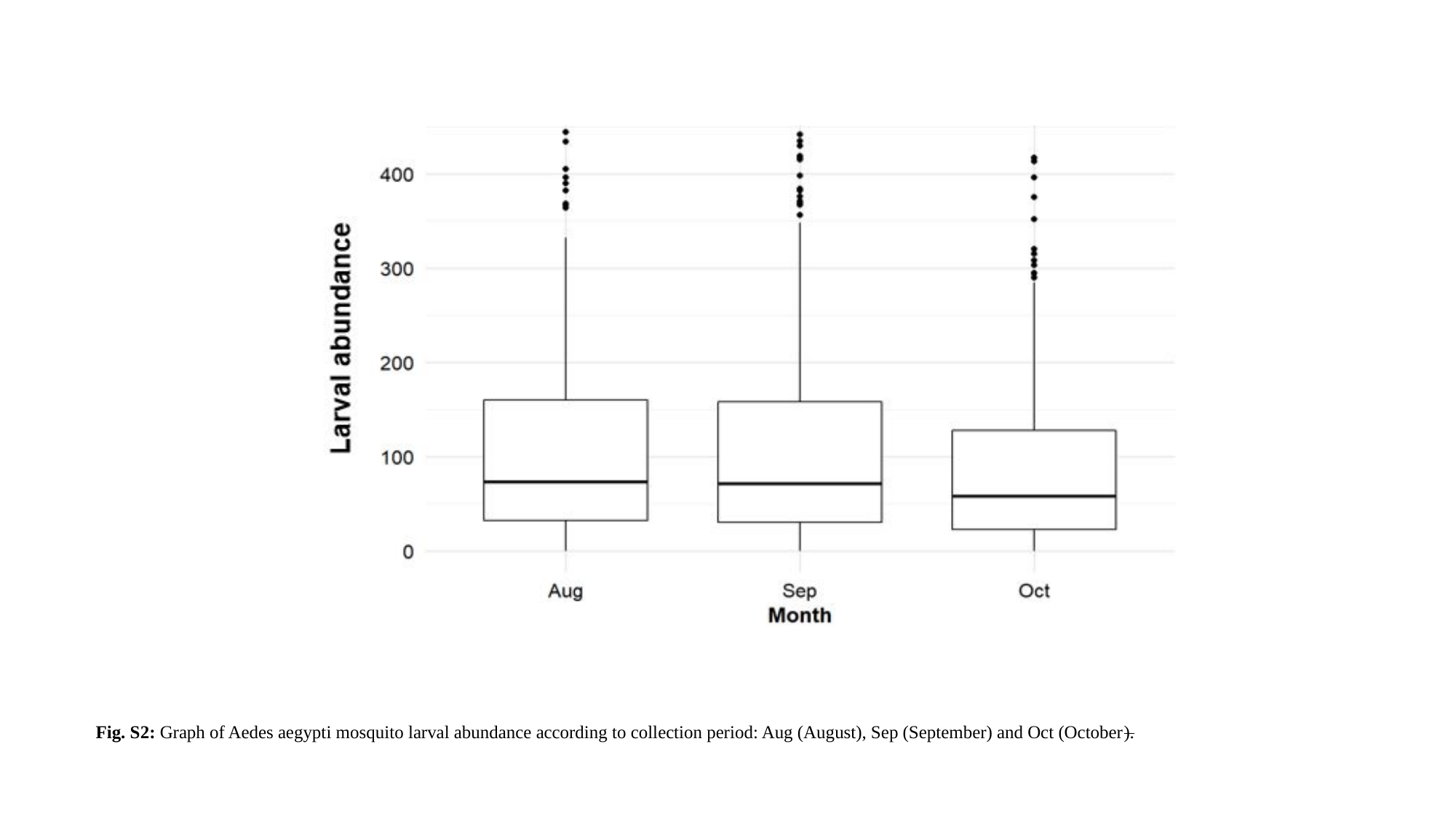

Fig. S2: Graph of Aedes aegypti mosquito larval abundance according to collection period: Aug (August), Sep (September) and Oct (October).

## Slide 3
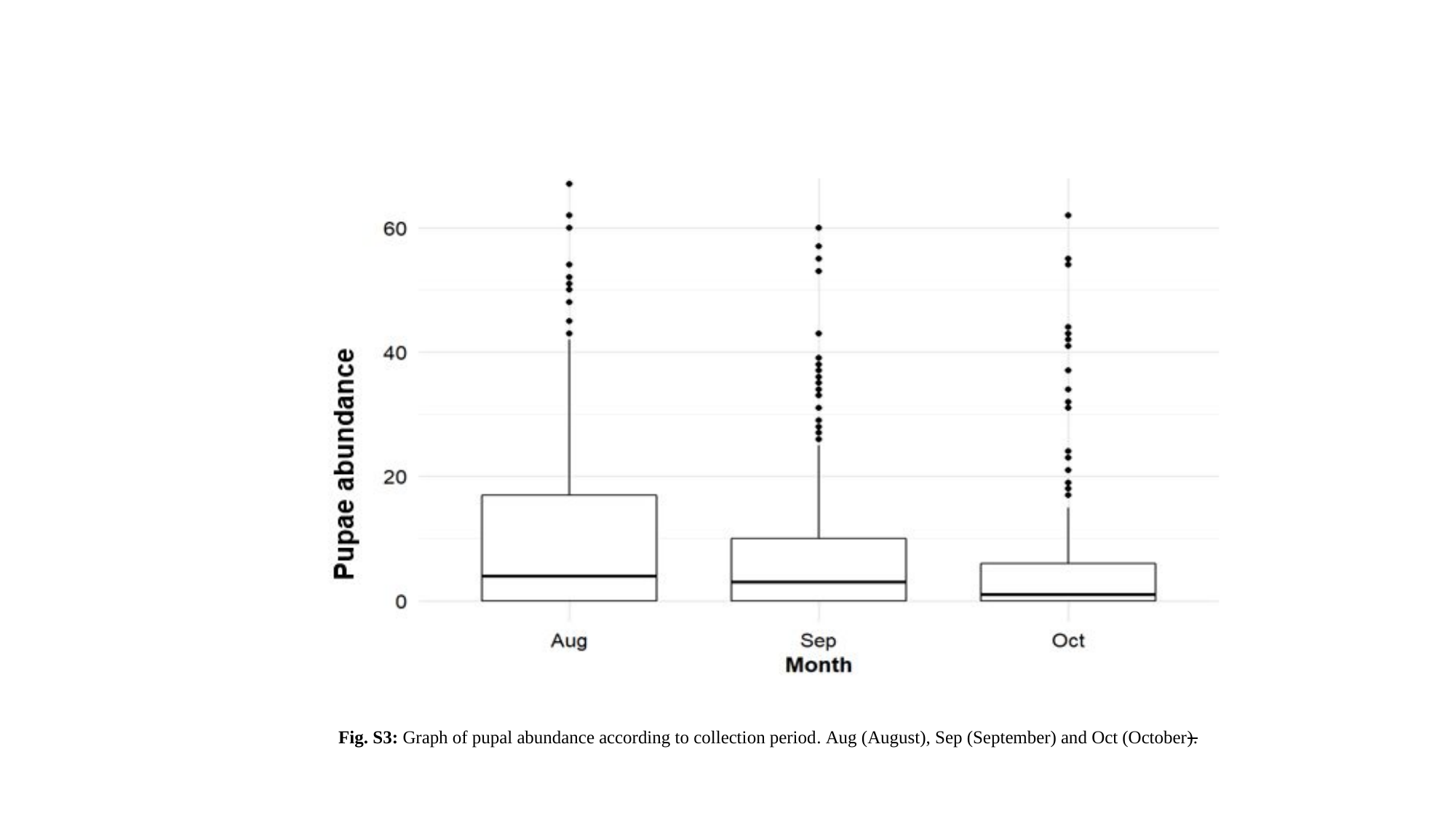

Fig. S3: Graph of pupal abundance according to collection period. Aug (August), Sep (September) and Oct (October).
